# High-Resolution ^18^F-FDG PET/CT for Assessing Three-Dimensional Intraoperative Margins Status in Malignancies of the Head and Neck, a Proof-of-Concept

**DOI:** 10.3390/jcm10163737

**Published:** 2021-08-22

**Authors:** Jens M. Debacker, Vanessa Schelfhout, Lieve Brochez, David Creytens, Yves D’Asseler, Philippe Deron, Vincent Keereman, Koen Van de Vijver, Christian Vanhove, Wouter Huvenne

**Affiliations:** 1 Department of Head and Skin, Ghent University, 9000 Ghent, Belgium; Lieve.Brochez@UGent.be (L.B.); Philippe.Deron@UZGent.be (P.D.); Wouter.Huvenne@UZGent.be (W.H.); 2 Department of Head and Neck Surgery, Ghent University Hospital, 9000 Ghent, Belgium; 3 Department of Nuclear Medicine, University Hospital Brussels, 1090 Brussels, Belgium; 4 Cancer Research Institute Ghent, 9000 Ghent, Belgium; Vanessa.Schelfhout@UZGent.be (V.S.); David.Creytens@UZGent.be (D.C.); Yves.DAsseler@UGent.be (Y.D.); Koen.VandeVijver@UZGent.be (K.V.d.V.); Christian.Vanhove@UGent.be (C.V.); 5 Department of Medical Imaging, Nuclear Medicine, Ghent University Hospital, 9000 Ghent, Belgium; 6 Department of Diagnostic Sciences, Ghent University, 9000 Ghent, Belgium; 7 Department of Dermatology, Ghent University Hospital, 9000 Ghent, Belgium; 8 Department of Pathology, Ghent University Hospital, 9000 Ghent, Belgium; 9 Department of Electronics and Information Systems, Ghent University, 9000 Ghent, Belgium; Vincent.Keereman@xeos.care; 10 XEOS Medical NV, 9000 Ghent, Belgium; 11 INFINITY Lab, Ghent University, 9000 Ghent, Belgium

**Keywords:** head and neck cancer, PET/CT, margin assessment, deep margins, molecular imaging

## Abstract

The surgical treatment of head and neck malignancies relies on the complete removal of tumoral tissue, while inadequate margins necessitate the use of adjuvant therapy. However, most positive margins are identified postoperatively as deep margins, and intraoperative identification of the deep positive margins could help achieve adequate surgical margins and decrease adjuvant therapies. To improve deep-margin identification, we investigated whether the use of high-resolution preclinical PET and CT could increase certainty about the surgical margins in three dimensions. Patients with a malignancy of the head and neck planned for surgical resection were administered a clinical activity of 4MBq/kg ^18^F-FDG approximately one hour prior to surgical initiation. Subsequently, the resected specimen was scanned with a micro-PET-CT imaging device, followed by histopathological assessment. Eight patients were included in the study and intraoperative PET/CT-imaging of 11 tumoral specimens and lymph nodes of three patients was performed. As a result of the increased resolution, differentiation between inflamed and dysplastic tissue versus malignant tissue was complicated in malignancies with increased peritumoral inflammation. The current technique allowed the three-dimensional delineation of ^18^F-FDG using submillimetric PET/CT imaging. While further optimization and patient stratification is required, clinical implementation could enable deep margin assessment in head and neck resection specimens.

## 1. Introduction

In times when systemic and radiotherapeutic approaches are constantly renewing, the surgical resection of solid malignancies remains the standard therapeutic approach, particularly in early stage malignancies of the head and neck [1]. Oncological surgeries in the head and neck are characterized by complex and invasive procedures, often requiring the excision of functional regions to ensure full oncologic removal. Dependent on the type and location of the tumor, a margin of ≥5mm needs to be obtained to ensure full oncological removal, regularly necessitating immediate reconstruction [2]. However, assessing the surgical margins in these malignancies still relies on the surgeon’s visual and tactile feedback, often supplemented with frozen sections of the regions at risk [3]. Unfortunately, the lack of the current sensitivity and specificity to distinguish malignant and benign tissue during surgery results in a limited 15–26% of oral squamous cell carcinomas (SCC) having adequate margins after surgery [4]. When close or positive margins <5mm are identified on histopathological analysis postoperatively, completion surgeries are often obsolete because of the need for immediate reconstructive surgery after tumor resection. Therefore, adjuvant therapies including radiotherapy, whether or not associated with concomitant systemic therapies, are required, as a positive surgical margin is associated with a significantly worse survival [5]. Moreover, these often preventable adjuvant therapies can result in important esthetic, social and functional deficits, severely impacting the quality of life for the patient [6,7].

While recent advances have been proposed to increase the accuracy of margin assessment in a multitude of tumor types, assessing the deep margins with sufficient accuracy remains an important limitation in most proposed techniques [8]. Over the last decade, several techniques that could potentially enable intraoperative margin assessment, such as fluorescence, hyperspectral imaging, spectroscopy and radioguided surgery, have been and still are being evaluated, with varying success [9,10,11,12]. More specifically, the use of fluorescent tumor-targeting tracers to delineate malignancies has become a growing topic of interest, with an expanding number of clinical trials in different malignancy types [13]. While preliminary results show that these techniques are able to visualize the malignancies in situ, their limited penetration depth remains a major limitation in visualizing the deep margins [3,14]. These limitations are especially important in malignancies of the head and neck, where 71.4–87% of all positive margins are deep surgical margins [2,15,16]. Increasing certainty about the deep margins is therefore of major importance to increase survival in this patient group.

As ^18^F-fluorodesoxyglucose (^18^F-FDG) remains the most accurate radiotracer in solid malignancies of the head and neck on clinical PET/CT, we hypothesized that ^18^F-FDG could be used as a contrast agent for the identification of a solid malignancy in resected specimens [17,18]. Fortunately, while even the best state-of-the-art clinical PET devices remain limited to a spatial resolution of approximately 3 mm, advances in preclinical imaging have resulted in the development of small high-resolution PET- and CT-imaging devices with a near five-fold optimization in spatial resolution as opposed to the standard clinical PET/CT devices [19,20]. Consequently, by using these small benchtop high-resolution PET/CT-imaging devices with tumor-specific radiotracers on resected specimens intraoperatively, the deep surgical margins could be visualized with an increased certainty, hence adapting the surgical approach resulting in a decrease in postoperative invaded margins.

In the current proof-of-concept study, we used a high-resolution benchtop preclinical PET/CT-imaging device to assess the margin status on surgically derived specimens from patients diagnosed with a malignancy of the head and neck.

## 2. Materials and Methods

The current prospective study was performed at a single tertiary reference center and was approved by the local committee for medical ethics (registration number 2019/1135) and the Belgian Federal Agency for Nuclear Control (FANC) prior to initiation. Patients were eligible for the study when they included all of the following criteria: (1) the patient is diagnosed with a histopathological-proven malignancy of the head and neck; (2) the patient was planned for surgical removal of this malignancy (3) the patient was not pregnant, nor breastfeeding at the time of injection (4) the patient has given informed consent to participate in the trial.

### 2.1. Study Flow

Patients were recruited at the department of Head and Neck surgery by a member of the study team. On the day of surgery, patients were brought to the department of Nuclear Medicine, where glycemia was measured prior to administering a clinical activity of 4MBq/kg ^18^F-FDG to the patient. The patient was brought to the operating room immediately after administration. In the operating room, surgery was performed as standard of care. To ensure safety for the health personnel, all members present in the operating room were obliged to wear a personal dosimeter during the full duration of the surgery. When a sample was excised by the surgeon, the sample was oriented using surgical sutures to ensure correct positioning. Afterwards, the sample was moved into a lead container to ensure safety for accidental passengers and brought to the PET/CT room.

### 2.2. Scanning

Prior to scanning the sample, the residual activity in the individual samples was measured using a VDC-404 dose calibrator (Veenstra Instruments, Joure, The Netherlands). After measuring the sample’s activity, the specimen was positioned on an imaging bed using the specimen’s surgical suture to guide orientation and a photo was taken of the sample. High-resolution PET and CT images were acquired using a Molecubes β-CUBE and X-CUBE (MOLECUBES NV, Belgium), respectively. First, a 30-min PET-acquisition was performed, followed by a (5-min) high-resolution CT-scan protocol. PET-CT images were reconstructed using MOLECUBES software. CT images were reconstructed with 200 µm voxel size using the Image Space Reconstruction Algorithm (ISRA). PET images were reconstructed using the Ordered Subset Expectation Maximization (OSEM) algorithm using 1 subset, 20 iterations, a voxel size of 800 μm, and an energy window of 50% around the 511 keV photopeak. In order to simulate a shortened PET acquisition time and lower administered ^18^F-FDG activity, a time frame of only the first few minutes of the original 30 min PET acquisition was reconstructed. A time frame of 7.5 min was used to simulate 1/4th of the administered dose acquired for 30 min, a time frame of 5 min was used to simulate an acquisition time of 5 min using the full dose, and a time frame of 1.25 min was used to simulate PET-scan with 1/4th of the administered dose and 5 min acquisition time. Data-analysis and post-processing were performed using A Medical Imaging Data Examiner (AMIDE), a Linux-based open source image data examiner [21].

### 2.3. Pathology

After imaging, the samples were brought to the department of Pathology, where a clinical pathologist inked the section margins as per local protocol. Slicing of the tissue was performed prior to specimen fixation, when possible. Specimens were fixed for 12–72 h in a 10% neutral buffered formalin solution. In order to correlate the pathological sections to the imaging, every slice was given a number and a photo was taken. Lymph nodes and samples derived from neck dissections were processed as standard of care. All samples were fixed for 12–72 h in a 10% neutral buffered formalin solution prior to tissue processing. Pathological assessment was performed using the in-house standard procedure and hematoxylin-eosin staining was performed on 4 µm slices. Additional immunohistochemical staining was performed, when required. In order to correlate the results of the imaging, these pathological reports were taken as gold standard and section margins on the pathological slices were correlated with the position of the section margins on PET/CT-imaging.

## 3. Results

**Figure 1:**
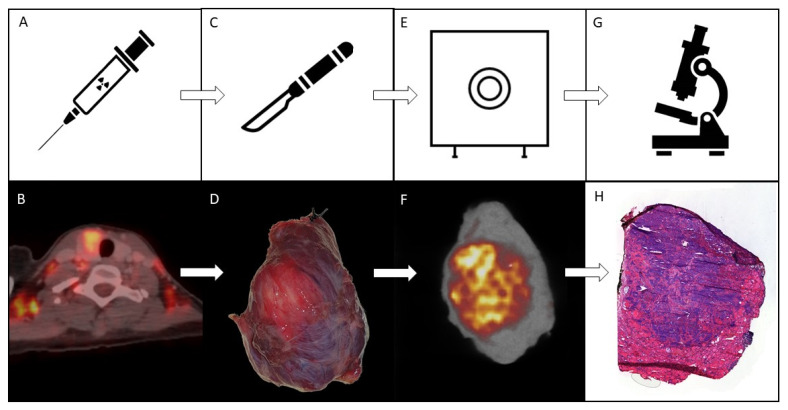
Schematic overview of the used methodology. The top row displays a simplified overview of the used methodology for the trial. The bottom row displays the step-by-step imaging results visualized in patient 7. (**A**): the patient is given 4 MBq/kg of ^18^F-FDG approximately on hour prior to planned surgical initiation. (**B**): Pretherapeutic clinical whole body ^18^F-FDG PET/CT taken during clinical routine staging. This image illustrates the increased ^18^F-FDG uptake in the left hemithyroid approximately 60 min after ^18^F-FDG administration. (**C**): Surgery is performed as standard of care. (**D**): Photographic image of the resected hemithyroid containing the solid malignant mass. (**E**): The resected specimen is scanned using preclinical high resolution PET- and CT-imaging devices. (**F**): High-resolution PET/CT-imaging of the full specimen displayed in (**D**). (**G**): When scanning is finalized, the specimen is processed for pathological assessment as standard of care, enabling margin assessment. (**H**): Hematoxylin-eosin staining of a pathological slide including both malignant and healthy tissue, characterized by more hematoxylin (purple) and eosin (pink) staining, respectively.

Between August 2020 and June 2021, we included a total of 8 patients into the trial, resulting in a total removal of 11 different tumor-containing specimens. A summary of the patient and tumor characteristics is found in Table 1. A figurative overview with results of patient 7 is given in Figure 1.

### 3.1. (Pre)Surgical Flow

After giving written informed consent, all included patients received an activity of ^18^F-FDG prior to surgical initiation. The average administered activity was 322 ± 74 MBq (range 221–478 MBq), which was administered on average 48 ± 20 min (range 20–99 min) prior to the start of the patient’s anesthesia (see Table 2). Surgical dissection and PET/CT scanning of the tumor specimen were performed on average 130 ± 22 min (range 74–169 min) and 139 ± 23 min (range 92–183 min) after ^18^F-FDG administration, respectively (see Table 2). This range in time was associated with the different surgical techniques required for the different malignancies, and the logistical challenges associated with the study protocol requiring a multidisciplinary cohesion of the study team. All surgeries were performed as standard of care.

### 3.2. Ex Vivo Specimen PET/CT

**Figure 2:**
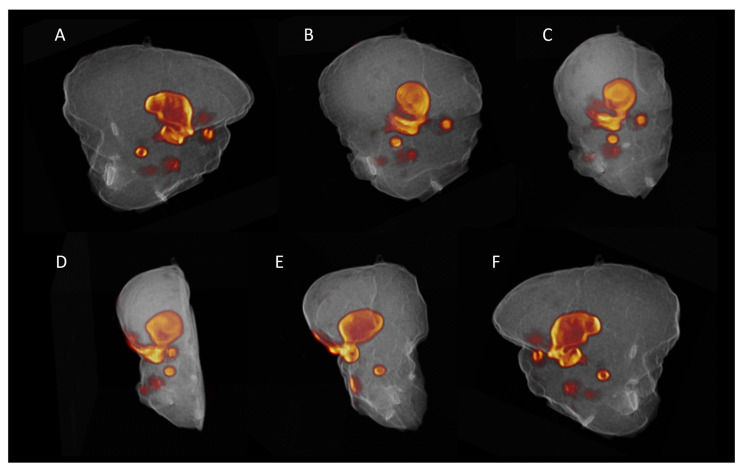
Three-dimensional render of the PET/CT-imaging results of the scanned specimen from patient 2. These images display a 3D-render of the imaging results collected by scanning a hemiglossectomy caused by a malignant squamous cell carcinoma in the tongue after ^18^F-FDG administration. From (**A**–**F**) the rendering is stepways rotated for 180° around the z-axis to provide full insight in the exact location of the ^18^F-FDG uptake within the scanned specimen. The imaging results hence display the proposed location of the malignancy and both superficial and deep tumor margins within the resected specimen. The full animation is provided in Supplementary Material S2.

As already mentioned, primary tumor specimens were scanned on average 139 min after initial injection of the patient using high-resolution PET/CT. One specimen (specimen 3) was cut by the responsible pathologist in two halves to fit on the imaging bed of the device. Prior to scanning the samples, specimen activity was measured in a dose calibrator and ranged from 48 to 3482 kBq (average 558 ± 1108 kBq). The exceptionally high activity measured in specimen one was found to be the result of the sentinel node procedure where ^99m^Tc that was injected peritumorally. Lymph nodes had a significantly lower residual activity with an average of 25 ± 12 kBq (range 10–47 kBq). Clear regions of increased ^18^F-FDG uptake could be identified in all specimens (Supplementary Material S1). Three-dimensional volume rendering of the PET/CT images resulted in a clear localization of the metabolic regions within the sample (Figure 2 and Supplementary Material S2).

### 3.3. Pathology and Correlation with the Imaging Results

**Figure 3:**
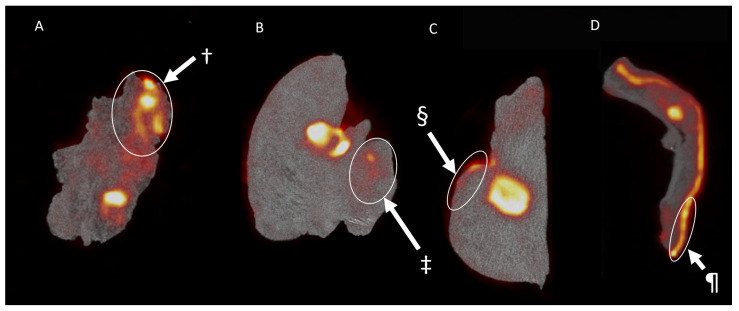
The ^18^F-FDG uptake in pathologically proven non-malignant regions. The images display regions of increased ^18^F-FDG uptake that were later pathologically proven to be non-malignant tissue. (**A**) displays the coronal view of the resected squamous cell carcinoma (SCC) of the floor of mouth in patient 1. Here, we see an increased uptake in a large region that was later shown to be highly active non-malignant inflamed tissue (†). (**B**,**C**) display the coronal and sagittal view, respectively, of the hemiglossectomy for a mucosal SCC in patient 2. Here, we see an increased uptake of a salivary gland (‡) and premalignant dysplastic tissue on the tongue’s epithelia (§). (**D**) displays the imaging results found after imaging a nasal resection specimen for an angiosarcoma of the nasal tip in patient 6. Here, ¶ displays an increased uptake in the subcutaneous nasal sebaceous glands.

**Figure 4:**
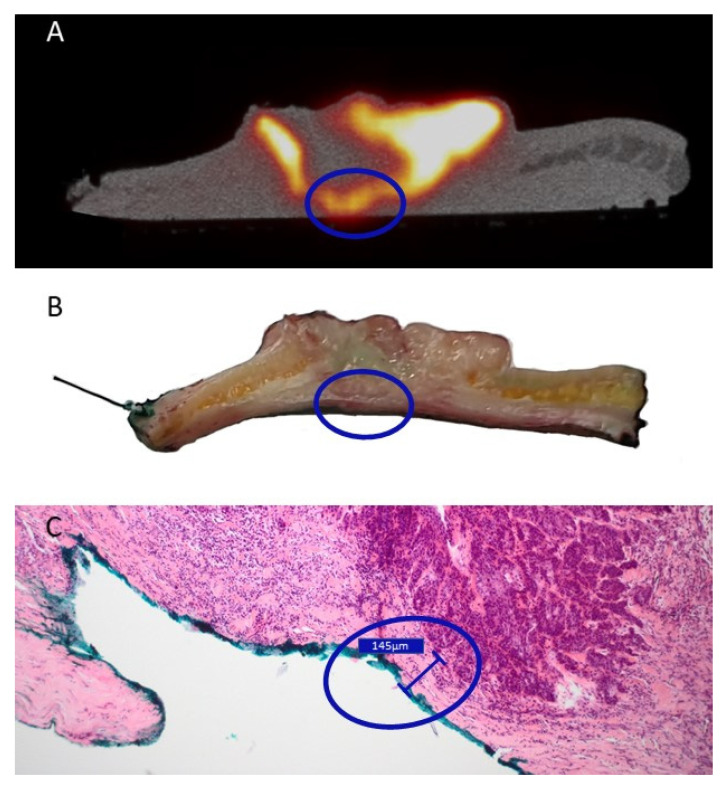
Correlation between the close margin as described during pathological assessment and PET/CT-imaging of a cutaneous squamous cell carcinoma of the scalp in patient 3. The blue circle encircles a region of interest that showed positive margin on PET/CT-imaging (**A**), ambiguous margin on the macroscopic pathological assessment (**B**), but was found to be a ‘close’ margin of 0.15 mm on final microscopical analysis (**C**). (**A**): Transversal view of PET/CT imaging of the resected specimen. (**B**): The sliced tissue of the proposed region corresponding with the PET/CT-imaging found in (**A**). (**C**): microscopic pathological assessment of the hematoxylin and eosin-stained slide made after fixation of the macroscopic slide displayed in (**B**).

All resected specimens were cut in parallel slices prior to pathological assessment. This enabled the macroscopic pathological identification of the ^18^F-FDG high-uptake regions. While coregistration was not fully accurate, this allowed a rough colocalization of the centrals slides of the tumor specimen and the PET/CT images. All macroscopic malignant regions could be correlated with regions of increased ^18^F-FDG uptake, displaying an excellent sensitivity for the technique. However, in two patients receiving the resection of a SCC of the oral cavity (patient 1 & patient 2), regions with increased ^18^F-FDG were associated with regions of inflammation (Figure 3A), salivary glands (Figure 3B) and dysplasia (Figure 3C). These regions of increased uptake were however not visible in the resected tongue tumor from patient 9 (Supplementary Material S1.11). Moreover, in patient 4, who received a total nasal resection for an angiosarcoma, diffuse ^18^F-FDG uptake could be identified (Figure 3D). Interestingly, besides the malignancy, the uptake could be correlated with an increased uptake in the sebaceous glands. In contrast, patient 3, who received the resection of a SCC of the scalp, was identified with a pathological close margin between the invasive tumor and the resection margin that was calculated to be 145 µm. As displayed in Figure 4, when correlating PET/CT-imaging and pathology, this region could be located on the imaging as a small region where the deep margin shows an increased ^18^F-FDG uptake.

As displayed in Supplementary Material S3, a 1/4th administered activity of ^18^F-FDG or a reduced scanning time of 5 min allow imaging with sufficient contrast to delineate high metabolic and low metabolic regions in the specimen in most specimens. However, the amount of noise increases with decreasing acquisition time or administered activity. Consequentially, emulating the imaging with 1MBq/kg of ^18^F-FDG and 5 min of PET-acquisition severely decreases contrast in most specimens.

### 3.4. Lymph Nodes

**Figure 5:**
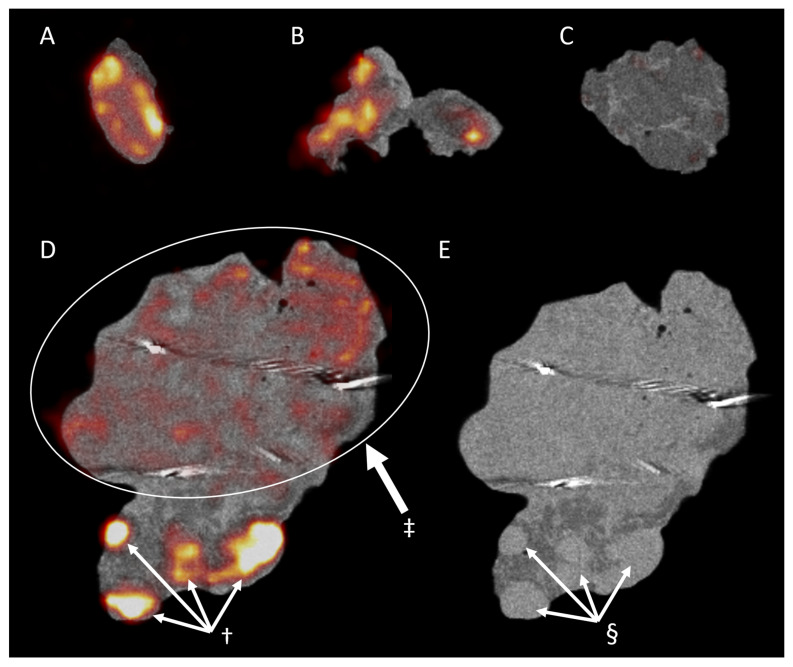
PET and CT-imaging of the resected lymph nodes. A and B display the ambiguous ^18^F-FDG uptake of non-malignant lymph nodes in contrast to C that eventually led out to be fatty tissue and could hence be used as a negative control. (**D**,**E**) display the PET/CT and CT-imaging, respectively, of the resected region 1a during neck dissection. These images display the presence of (non-malignant) lymph nodes both on PET (†) and CT (§). Moreover, ‡ displays the physiological ^18^F-FDG uptake in the submandibular salivary gland (**A**): Negative sentinel lymph node of patient 1. (**B**): Two negative sentinel lymph nodes of patient 1. (**C**): Fatty tissue that was resected during sentinel procedure in patient 1. (**D**): PET/CT imaging of the anatomical neck region 1a resected during lymphadenectomy from patient 2. (**E**): CT-imaging of anatomical neck region 1a resected during lymphadenectomy from patient 2.

In patients 1, 2, and 9, lymph nodes were resected and scanned separately from the tumor specimen. Patient 1 received a sentinel lymph node procedure with preoperative administration of ^99m^Tc-nanocoll, followed by Single Photon Emission Computed Tomography (SPECT), CT and intraoperative node identification using a gamma probe (Neoprobe, Devicor Medical Products, Inc., Cincinnati, USA). The ^18^F-FDG was administered after SPECT/CT-imaging, which resulted in a significant amount of background noise on the gamma probe as a result of the gamma-radiation of the ^18^F-FDG. This made intraoperative identification of the nodes cumbersome, and surgical guidance with the SPECT/CT was required to identify the nodes (*n* = 3), which were scanned using the high-resolution PET/CT system prior to pathological assessment (Figure 5). Two of the three specimens displayed a clear ^18^F-FDG uptake, while one displayed nearly no ^18^F-FDG uptake. Interestingly, during pathological assessment, the ^18^F-FDG negative specimen (Figure 5C) included only fat tissue, while the two other specimens included one (Figure 5A) and two (Figure 5B) lymph nodes without identifiable malignancy. Patients 2 and 9 received an elective unilateral functional neck dissection. The resected neck regions were also scanned following the tumor resection. Figure 5D displays the PET/CT results for the resected lymphadenectomy from patient 2. On these images, a high ^18^F-FDG uptake in multiple delineated regions could be identified, that also appear denser on the CT images (Figure 5E). On pathological assessment these high-uptake regions were found to be lymph nodes, which were all negative for malignancy. In the same resected specimen, a large region of heterogeneous uptake without a single peak was imaged (Figure 5D). This region of heterogeneous ^18^F-FDG uptake delineates the submandibular salivary gland.

## 4. Discussion

There is an urgent need for novel techniques that improve intraoperative decision making by obtaining the margin status with sufficient reliability and robustness, while remaining feasible for implementation in a clinical routine. To fulfill these requirements, we proposed the use of a small benchtop preclinical PET/CT scanner to image the malignant tissue within the resected specimen sample using a patient dose of 4 MBq/kg ^18^F-FDG as the tumor-targeting tracer. ^18^F-FDG was chosen for this purpose, as it remains the most sensitive clinically-available PET-tracer in head and neck SCC to date [22]. In our results, all scanned samples displayed an increased ^18^F-FDG uptake on the macroscopically confirmed malignant regions, enabling the intra-operative three-dimensional visualization of the malignancy within the resected tissue. Consequentially, we here display an important proof-of-concept that displays the feasibility of using high-resolution PET/CT on excised tumoral tissue to assess both superficial and deep margins in resected malignancies of the head and neck.

In contrast to modern clinical-grade PET-devices that have a spatial resolution of approximately 3–5 mm, dependent on the type and brand, the preclinical PET device used for this trial has a spatial resolution below 1 mm [23]. Because of this previously unprecedented resolution, we were able to visualize an important heterogeneity in two patients with mucosal SCC (patients 1 & 2) and one patient with nasal angiosarcoma (patient 4) on this submillimetric scale. The two patients with mucosal SCC were p16-negative, proposedly co-initiated by tobacco and alcohol consumption. The heterogeneous uptake was assumed to be the result of false-positive peri-tumoral inflammation, premalignant dysplasia and sebaceous glands, that were confirmed during histopathological analysis [24]. Interestingly, we also included a rare case of a 28-year-old female without any risk factors with a p16-negative SCC of the tongue. On PET/CT-imaging, this specimen displayed much less uptake on the normal epithelia of the tongue. However, we were unable to divide inflammatory tissue and malignant tissue with absolute certainty by solely using the PET/CT-imaging. Similarly, in cutaneous SCC, basocellular carcinoma (BCC) and the thyroid carcinoma, less heterogeneity was obtained. These results could indicate an important difference in peri-tumoral ^18^F-FDG uptake, dependent on the tumor type, origin and location. Further research on the correlation between the uptake of ^18^F-FDG uptake and its corresponding histology will increase our understanding of (peri-)tumoral ^18^F-FDG distribution, and could hence help in correctly identifying and staging malignancies in head and neck and the thyroid on clinical PET/CT [25,26].

Interestingly, the heterogeneity of submillimetric ^18^F-FDG assessment is continued in the physiological uptake of benign lymph nodes. All analyzed lymph nodes were easily identifiable on high-resolution PET, but none eventually contained malignant tumor. Because no samples of invaded lymph nodes were scanned, we were unable to compare uptake in malignant and benign lymph nodes. While ^18^F-FDG PET/CT is one of the best staging methods in SCC of the head and neck (HNSCC), these results contain important information on the uptake of ^18^F-FDG in benign cervical lymph nodes [27]. This important physiological uptake is a typical pitfall in clinical PET/CT and hence stresses the important ambiguity in differentiating between benign and malignant cervical lymph nodes in patients diagnosed with head and neck cancer based on their PET/CT [24]. This ambiguity is emphasized by the results of a recent large multicenter study where, on visual reading of ^18^F-FDG PET/CT in the clinically N0 neck of patients with HNSCC, a negative predictive value of 0.87 was associated with a limited positive predictive value of only 0.44 [28]. More in depth research on the specific uptake in benign and malignant cervical lymph nodes could help in the future clinical staging of patients with HNSCC.

The proposed technique to assess tumoral margins is one of many novel techniques that have been proposed to increase the intraoperative identification of malignant tumor [29]. However, these techniques are characterized by inherent limitations, of which one recurring limitation is the limited penetration depth resulting in a limited assessment of the deep resection margins [8]. This lack of three-dimensional insight results in a difficult identification of the malignant tumor with sufficient accuracy and certainty. While the here-described methodology has no impact on the in situ identification of malignant tumor, it does demonstrate a possible solution to assess the malignant tumor within the resected specimen ex vivo with increased certainty. In contrast to e.g., breast cancers, where a negative margin is defined as no tumor on the inked specimen edge, multiple cancers, including HNSCC, require a margin of preferably five millimeters around the tumor [2,30]. To assess these deep margins, a thorough understanding of the signal depth is required, which might be missed using superficial imaging-techniques, but could be visualized using high resolution PET/CT.

Optimizing the correlation of the PET imaging with histopathology could increase our current understanding of ^18^F-FDG distribution in and around human malignancies. After all, it is known that ^18^F-FDG might be suboptimal in identifying margin status with sufficient certainty in mucosal malignancies of the head and neck. Our results display the low specificity and consequential uncertainty of using a metabolic tracer such as ^18^F-FDG for margin assessment in HNSCC. Using more tumor-specific PET-tracers, such as the prostate specific membrane antigen (PSMA) in prostate cancer [31], or more recently fibroblast activation protein inhibitor (FAPI)-targeting agents [32], could significantly increase both the specificity and sensitivity of the technique. Moreover, recent evolutions in translational nuclear medicine resulted in the development of repurposed hybrid fluorescent and radionuclide tracers which are revolutionizing the sentinel node procedure [33,34], and novel tumor-targeting hybrid tracers are being developed in preclinical and ex vivo clinical trials [35,36,37]. These hybrid tracers display the possible synergistic value of using both radionuclides and fluorescence intraoperatively to assess the margins in situ, using fluorescent imaging to plan and perform the surgery, supplemented with ex vivo PET/CT of the resected specimen to image the margins in three dimensions. Not only can positive section margins be visualized, the complete tumor is visualized within the resected specimen.

However, an important requirement for possible clinical implementation is the technique’s logistic feasibility. As this is a rather complex and inherent multidisciplinary technique, efficient communication between all medical departments needs to be guaranteed. The current study protocol takes over 40 min from excision to completion of scanning, not including the time needed to deliver the sample from the operating theater to the scanner room. As the current study protocol did not require immediate feedback to the surgeon, we used a rather long scanning protocol to ensure sufficient data, but as displayed in Supplementary Material S3, activity reduction and decreased scanning time allow sufficient imaging contrast when balanced correctly. Protocol optimization for clinical settings by reducing PET-acquisition time and relocating the PET/CT devices closer to the operating room could decrease the necessary time to finish the protocol. Moreover, with an optimized protocol, the PET/CT images could be available during the surgical procedure. While the proposed technique will not immediately replace the gold standard of histopathology, it could potentially enable the intra-operative identification of both superficial and close margins at risk, hence guiding the surgeon to these regions at risk for further pathological analysis.

We do acknowledge some limitations in the current trial. Because this was a proof-of-concept study, we included only a limited and heterogeneous group of patients, making statistical analysis impossible. Further investigation in specific tumor types could verify our results. While we were able to visualize ^18^F-FDG uptake in all scanned specimens, we acknowledge the inherent limitations of the tracer itself. Moreover, the difficulty to co-register the PET-images with the histopathology limits the exact correlation between PET-imaging and the pathological results with sufficient accuracy. Improving the co-registration between PET-imaging and histopathology would ensure the direct correlation between the imaging and pathological results. When direct co-registration could be obtained, the accurate values for sensitivity and specificity of ^18^F-FDG PET/CT could be obtained on this submillimetric scale.

## 5. Conclusions

In the current proof-of-concept study, we analyzed the capacity of a submillimetric PET/CT to assess ^18^F-FDG distribution in resected surgical samples from patients diagnosed with cancers of the head and neck. To the best of our knowledge, the current study is the first to use high-resolution ^18^F-FDG-PET/CT for assessing tumoral margins in resected tissue of the head and neck. These results display the feasibility and proof-of-concept of high-resolution PET to image ^18^F-labelled radionuclides in resected surgical samples in the head and neck. The findings of the current trial show the potential of ex vivo PET-imaging in margin assessment of head and neck resection specimens, but also in a more in-depth understanding of ^18^F-FDG uptake in malignancies.

## Figures and Tables

**Table 1:** Patient characteristics.

Patient Number	Age	Gender	Tumor Location	Tumor Type	Administered Activity (MBq)	Specimen Activity (kBq)
1	60	Male	Floor of mouth	SCC	297	3482
2	54	Male	Tongue	SCC	300	137
3	78	Male	Scalp	SCC	479	310
4	62	Male	Nose	Angiosarcoma	306	215
5	79	Male	Scalp	SCC	377	90
			Preauricular	SCC		
			Preauricular	BCC		
6	85	Male	Ear	SCC	340	94
			Preauricular	SCC		
7	28	Female	Thyroid	Medullary Carcinoma	257	85
8	60	Female	Tongue	SCC	221	48

SCC: Squamous cell carcinoma; BCC: Basocellular carcinoma.

**Table 2:** Necessitated time from administration to intermediate steps.

Patient Number	Specimen Number	Time of Anesthesia after Injection (min) ^A^	Time of Resection (min) ^B^	Time of PET/CT (min) ^C^
1	1	32	132	146
2	2	20	129	135
3	3	77	137	179
4	4	34	74	92
5	5	99	139	183
	6		159	
	7		169	
6	8	40	80	98
	9		165	
7	10	50	130	140
8	11	33	115	137
Average		48	130	139

^A^: The time between the administration of the ^18^F-FDG at the department of Nuclear Medicine, and anesthetic initiation. ^B^: The time between the administration of the ^18^F-FDG at the department of Nuclear Medicine and full resection of the respective specimen. ^C^: The time between the administration of the ^18^F-FDG at the department of Nuclear Medicine, and the start of PET-scanning of the specimen has started.

## Supplementary Materials

The following are available online at https://www.mdpi.com/article/10.3390/jcm10163737/s1, Supplementary Material S1: PET/CT-imaging of the surgical specimens, Supplementary Material S2: 3D video render of the PET/CT-imaging of specimen 2, Supplementary Material S3: Simulation of PET-acquisition with lower administered ^18^F-FDG and shorter scanning protocol.
