# Supplementary material for: High-Resolution 18F-FDG PET/CT for Assessing Three-Dimensional Intraoperative Margins Status in Malignancies of the Head and Neck, a Proof-of-Concept
Source: J Clin Med. 2021 Aug 22;10(16):3737. doi: 10.3390/jcm10163737 (PMC8397229; doi:10.3390/jcm10163737)
Supplement: Supplementary file 1 [file jcm-10-03737-s001.zip › jcm-1357371-supplementary.pdf]

Supplementary Material 1. PET/CT-imaging of the surgical specimens

Specimen number	White light picture of the resected specimen	PET/CT-image of the resected specimen
1	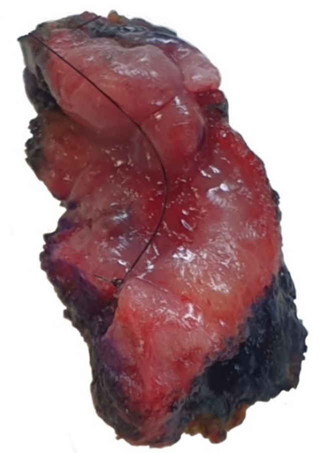	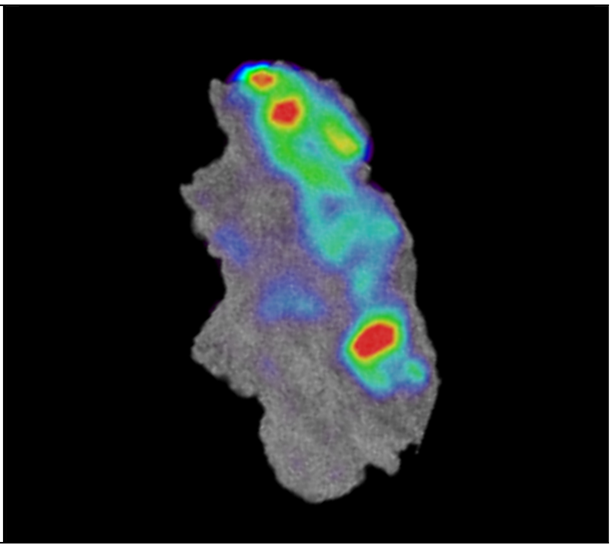
	Patient 1: Mucosal squamous cell carcinoma of the floor of mouth.	
2	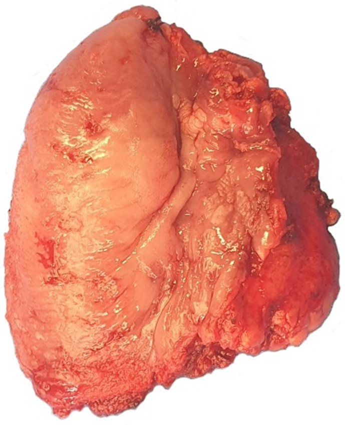	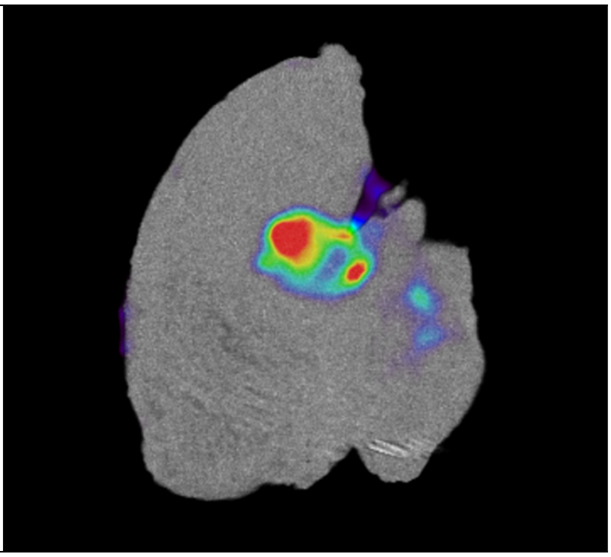
	Patient 2: Mucosal squamous cell carcinoma of the tongue.	
3	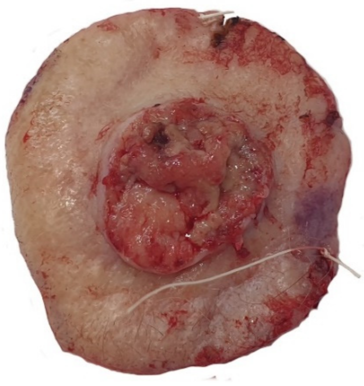	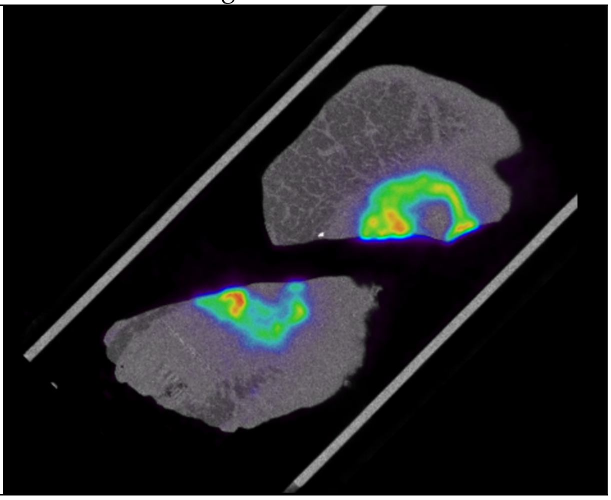
	Patient 3: Cutaneous squamous cell carcinoma of the scalp.	

4

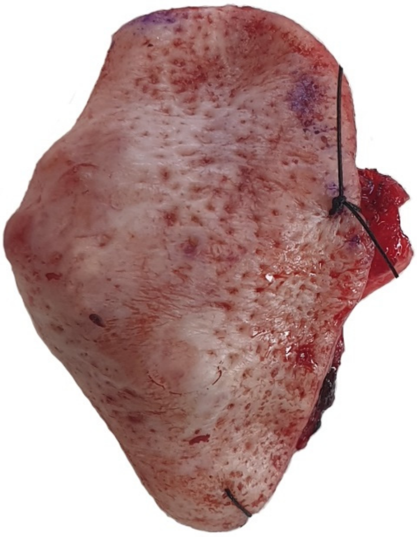

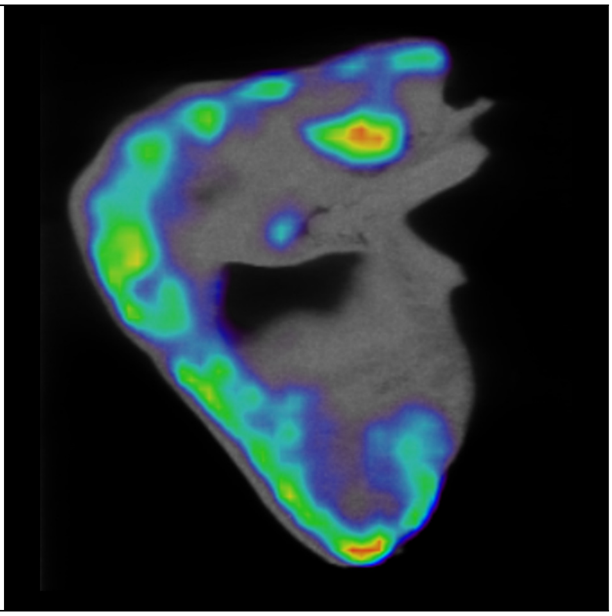

Patient 4: Angiosarcoma of the nasal tip.

5

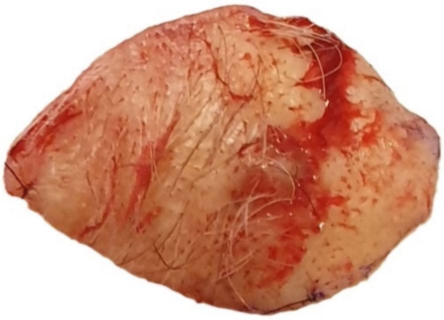

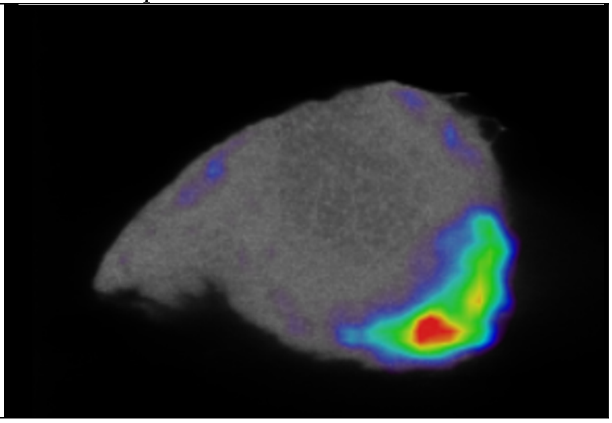

Patient 5: Cutaneous preauricular squamous cell carcinoma.

6

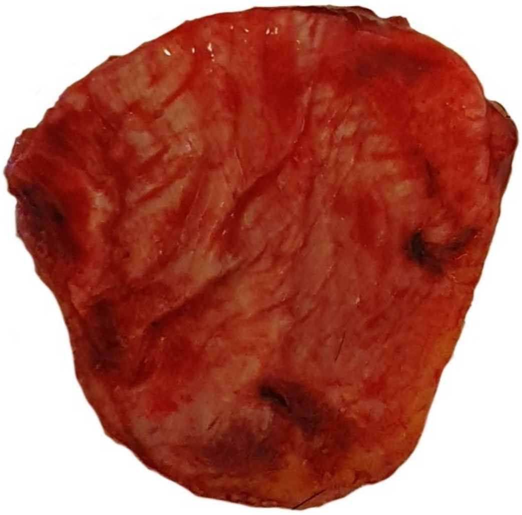

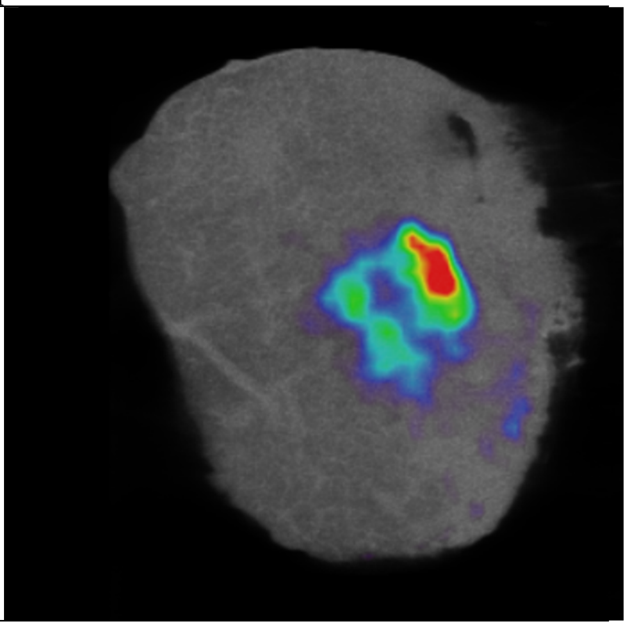

Patient 5: Cutaneous squamous cell carcinoma located on the scalp.

7	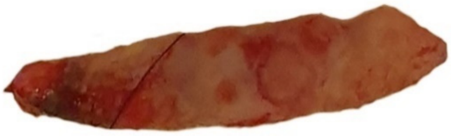	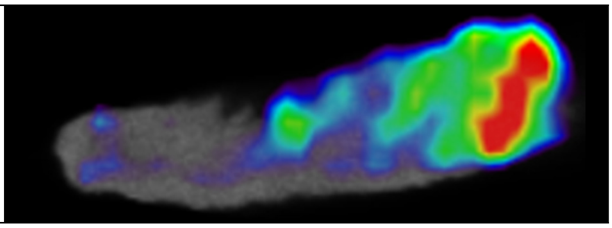
Patient 5: Cutaneous preauricular basocellular carcinoma .		
8	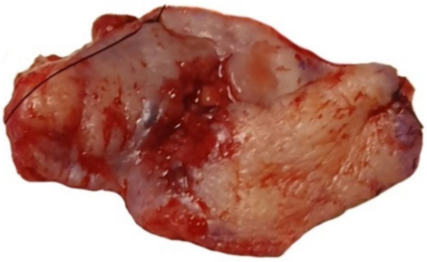	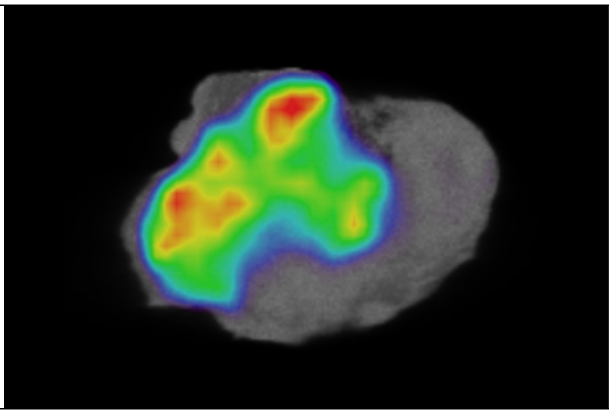
Patient 6: Cutaneous squamous cell carcinoma located on the external ear		
9	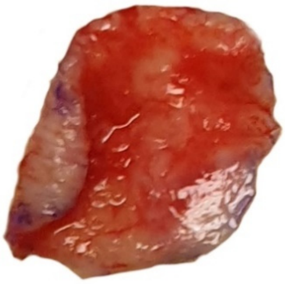	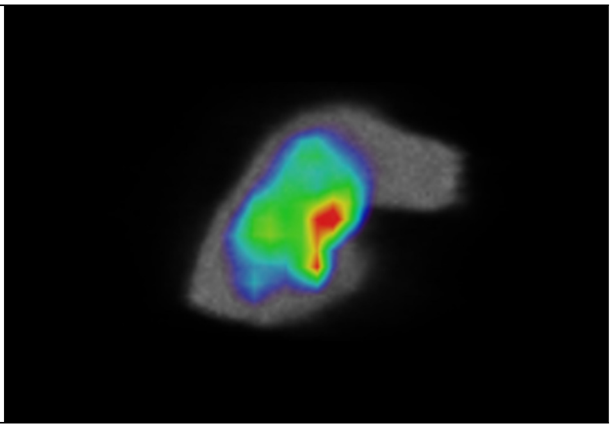
Patient 6: Cutaneous preauricular squamous cell carcinoma.		
10	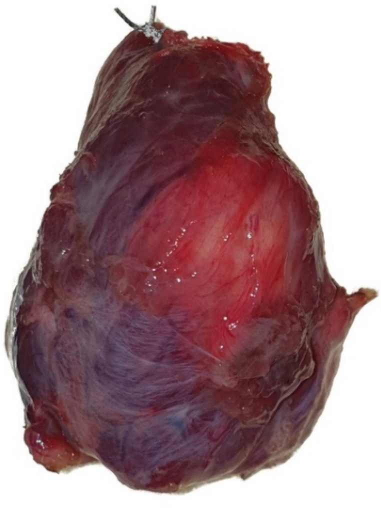	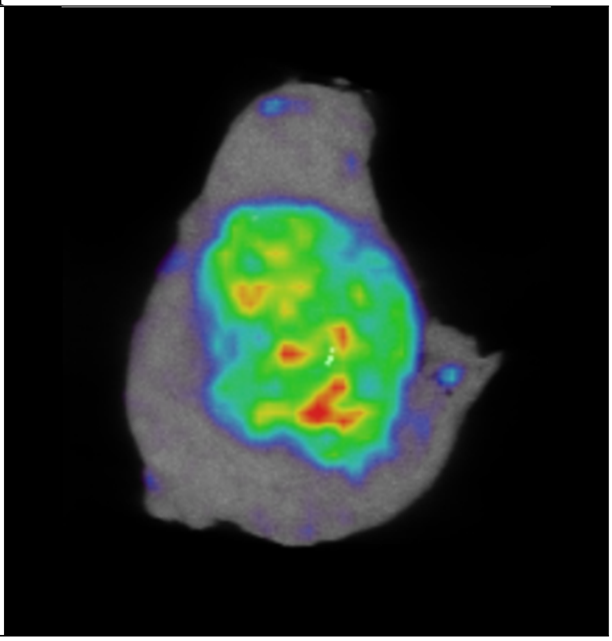
Patient 7: Medullary thyroid carcinoma.		

11	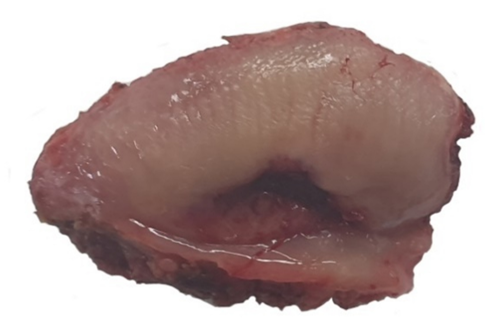	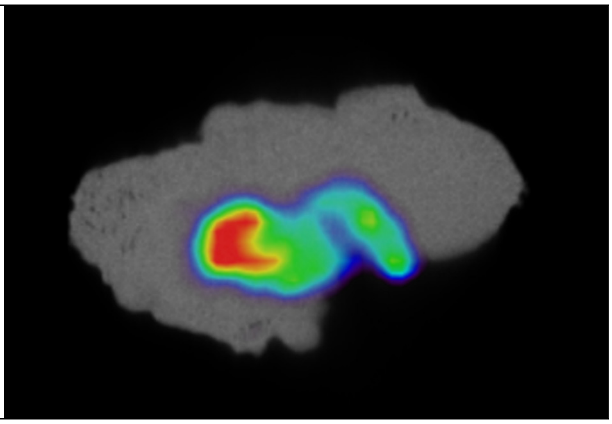

Patient 8: Mucosal Squamous cell carcinoma of the tongue.

Supplementary Material 2: 3D video render of the PET/CT-imaging of specimen 2

Supplementary Material 3. Simulation of PET-acquisition with lower administered ^{18}F-FDG and shorter scanning protocol

For this study, patients were injected with approximately 4MBq/kg of ^{18}F-FDG. The standard PET-acquisition time of the surgical specimens was always kept on 30 minutes for every scan. In order to simulate PET-scans with a lower acquisition time and a lower administered activity, a shorter time frame of only the first few minutes of the original PET acquisition was reconstructed. To simulate an administered dose of 1MBq/kg, using an acquisition time of 30 minutes, a time frame of 7.5 minutes was reconstructed. A time frame of 5 minutes was reconstructed to simulate a lower acquisition time of 5 minutes using a dose of 4MBq/kg. To simulate PET data using 1/4th of the activity and acquired during 5 minutes, a time frame of 1.25 minutes was reconstructed from the original 30 minutes PET-scan. The length of the time frames used during reconstruction are summarized in Table S1. For each tumoral specimen, a snapshot was taken with the different image reconstructions. The reconstructed images are provided in Figures S1–S11.

Table S1. Length of the reconstructed time frame used to simulate the decreased activity and acquisition time.

Simulated activity	Simulated acquisition time	Length time frame during reconstruction
Baseline (4MBq/kg)	30 minutes	30 minutes
¼ activity (1MBq/kg)	30 minutes	7.5 minutes
Baseline (4MBq/kg)	5 minutes	5 minutes
¼ activity (1MBq/kg)	5 minutes	1.25 minutes

	30′	5′
Baseline activity	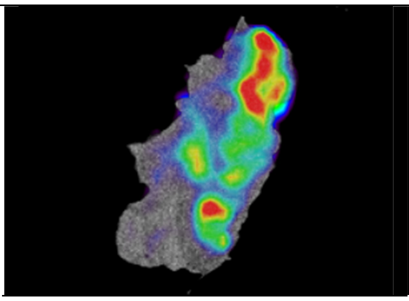	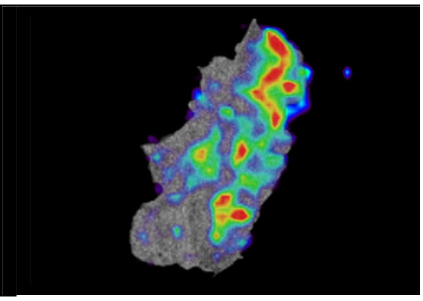

1/4th administered activity

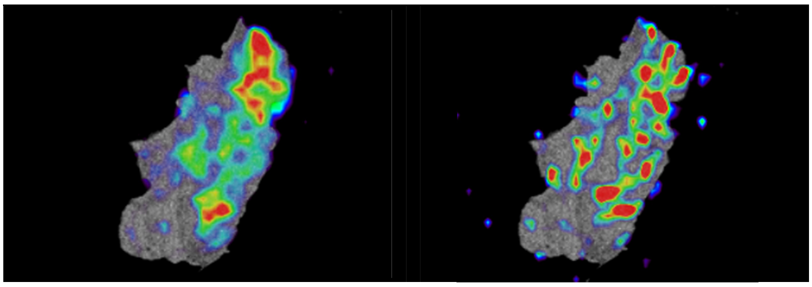

Figure S1. Original (top left) and simulated PET scans for specimen 1.

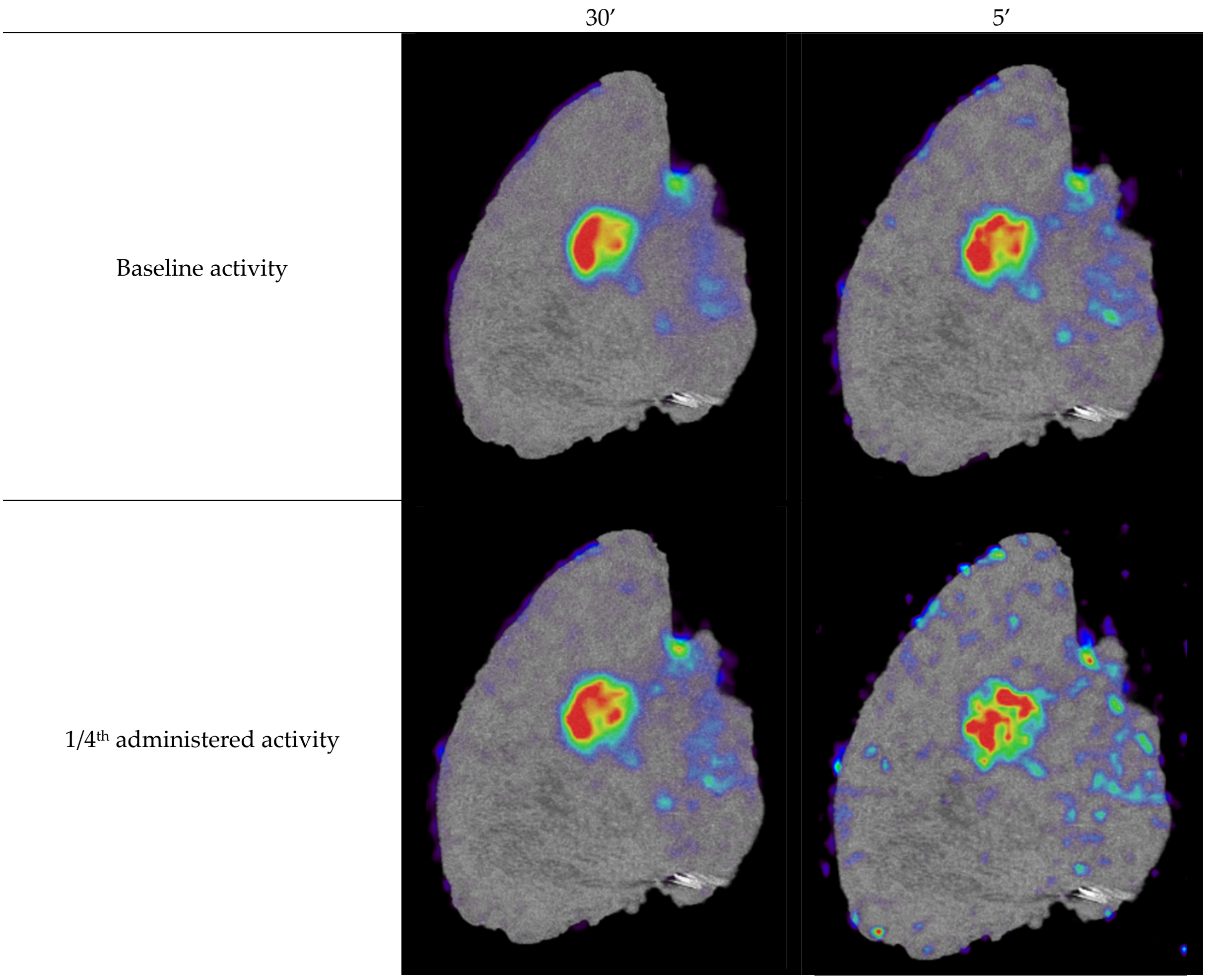

Figure S2. Original (top left) and simulated PET scans for specimen 2.

	30′	5′
Baseline activity	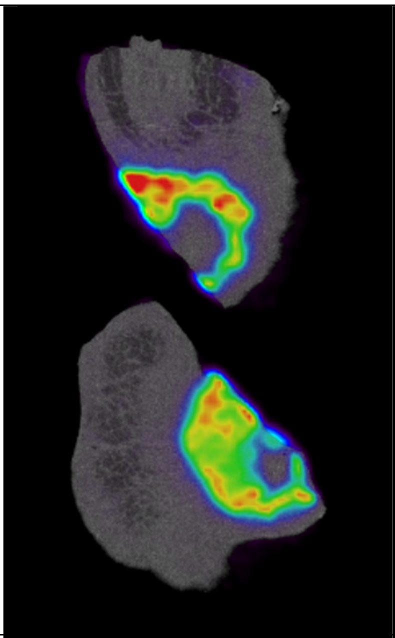	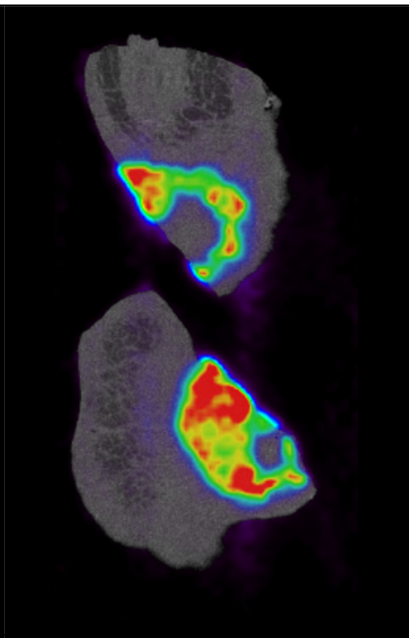
$1/4^{th}$ administered activity	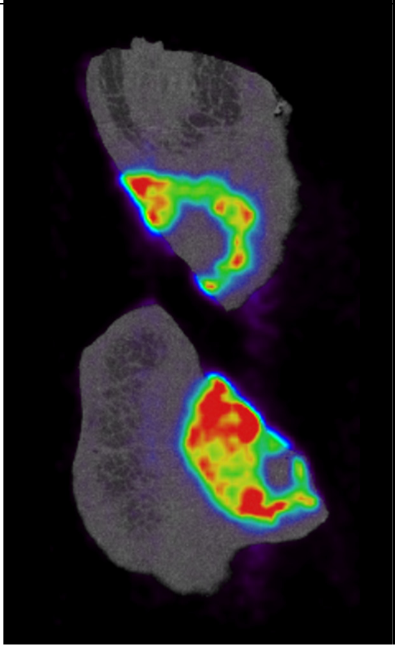	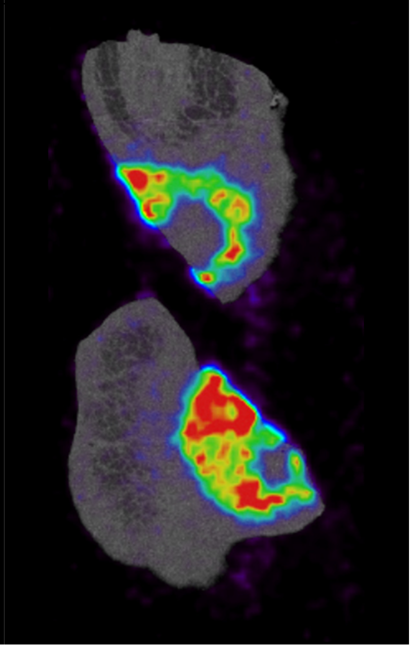

Figure S3. Original (top left) and simulated PET scans for specimen 3.

	30′	5′

Baseline activity

1/4th administered activity

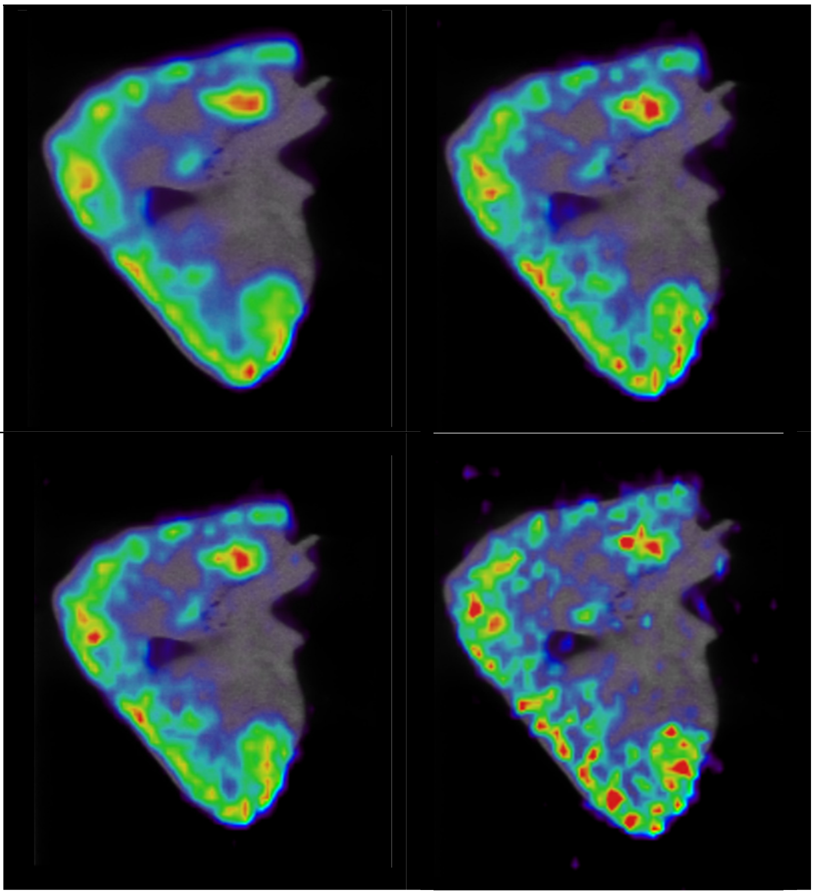

Figure S4. Original (top left) and simulated PET scans for specimen 4.

30′ 5′

Baseline activity

1/4th administered activity

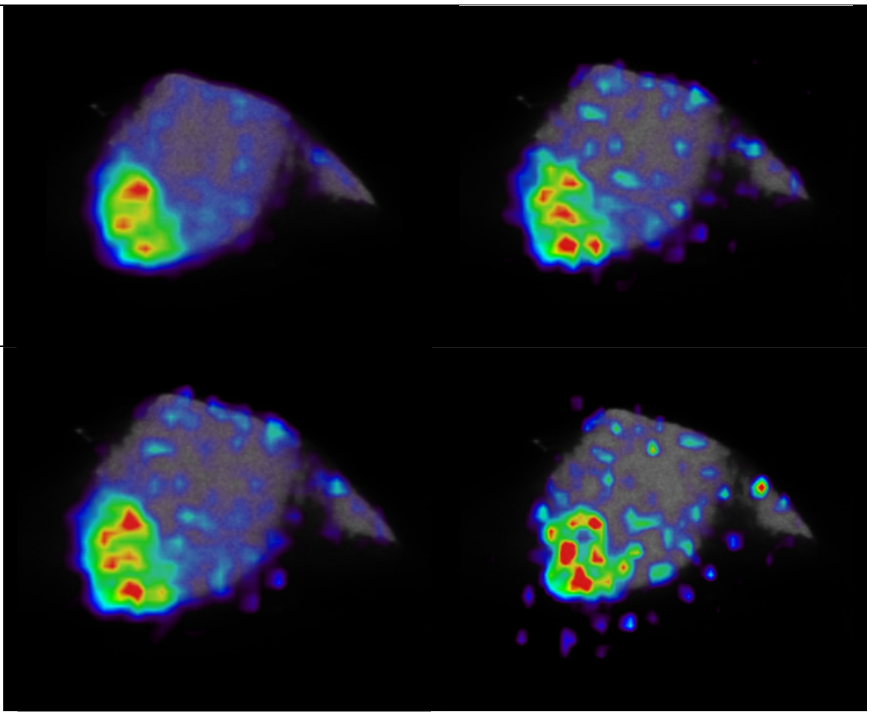

Figure S5. Original (top left) and simulated PET scans for specimen 5.

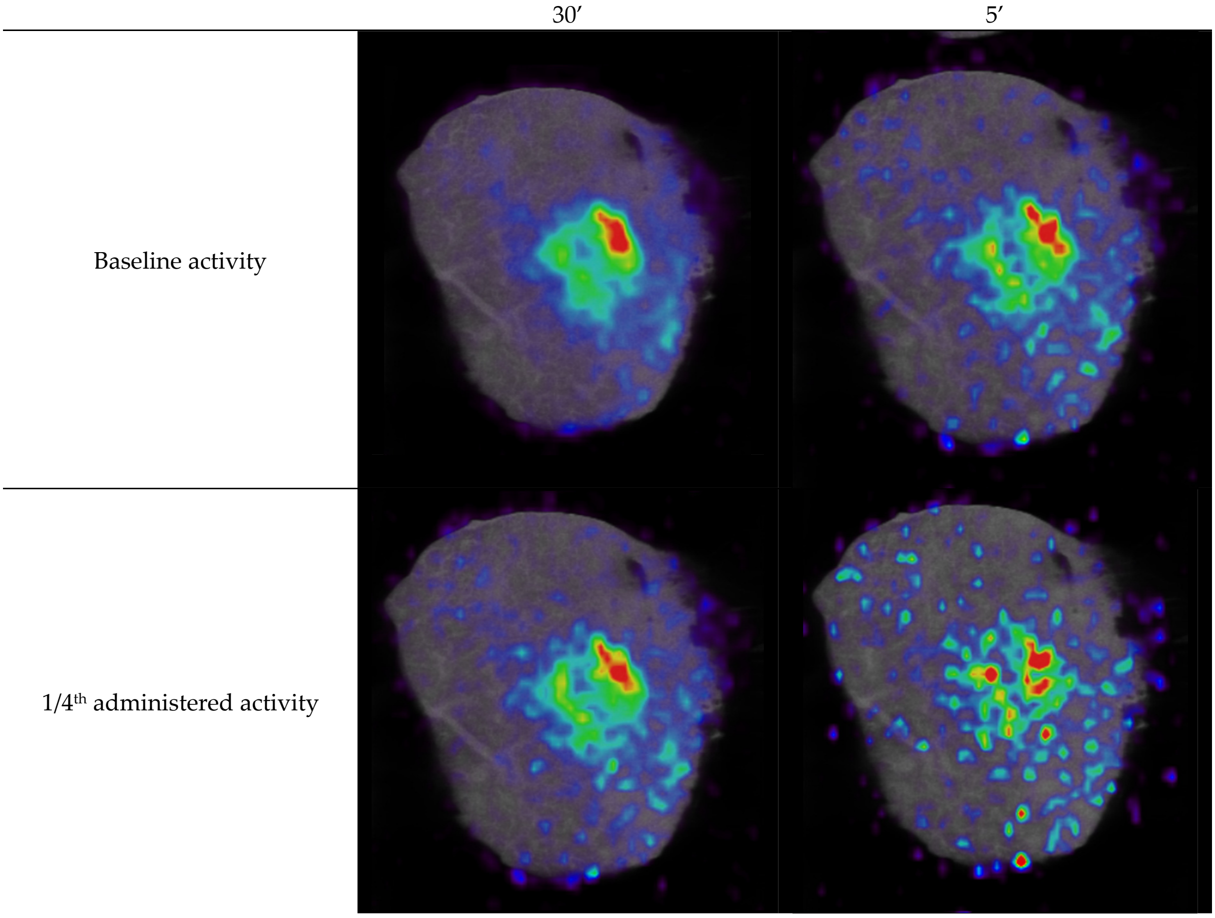

Figure S6. Original (top left) and simulated PET scans for specimen 6.

Specimen 7

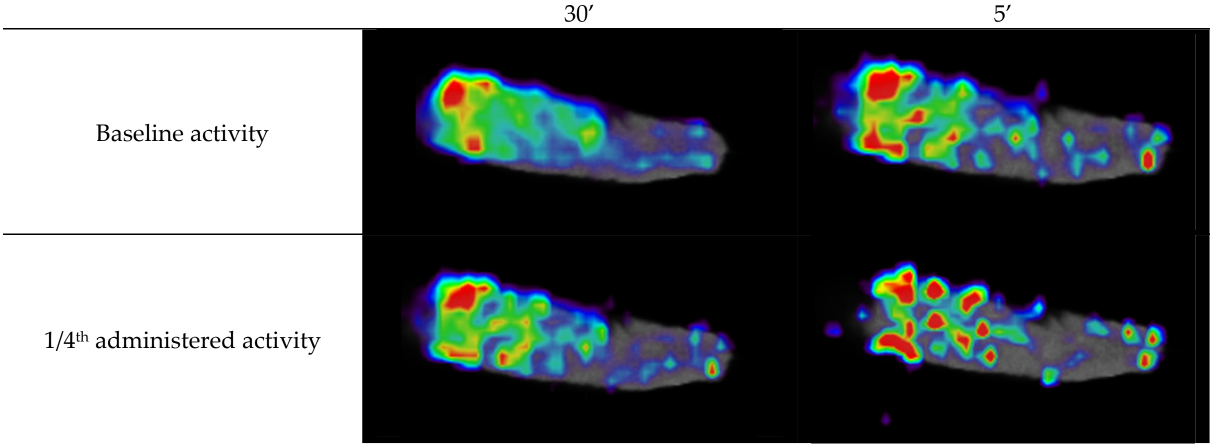

Figure S7. Original (top left) and simulated PET scans for specimen 7.

	30′	5′
Baseline activity	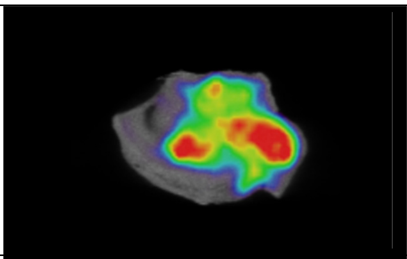	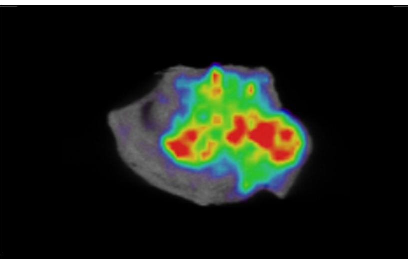
1/4th administered activity	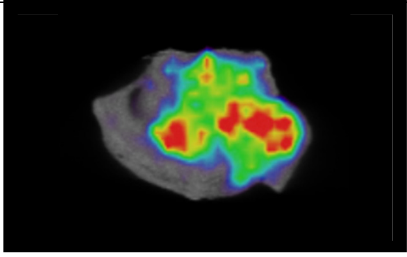	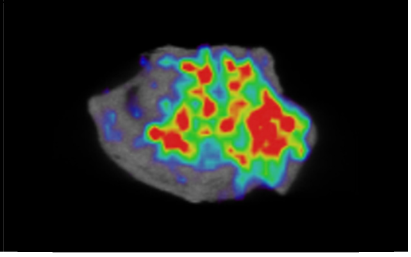

Figure S8. Original (top left) and simulated PET scans for specimen 8.

	30′	5′
Baseline activity	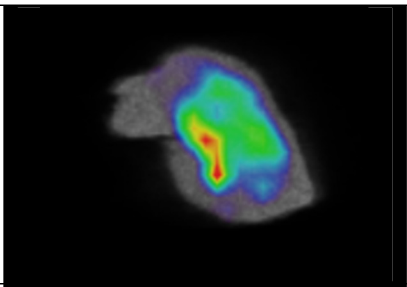	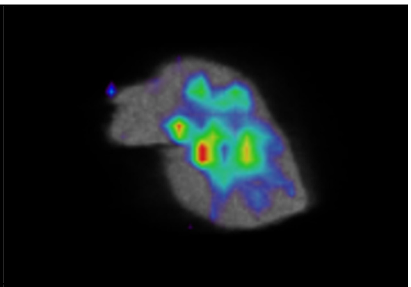
1/4th administered activity	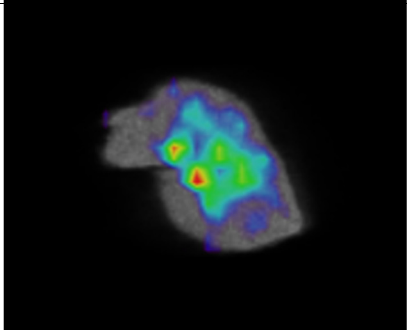	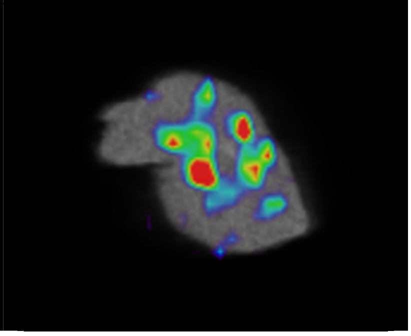

Figure S9. Original (top left) and simulated PET scans for specimen 9.

Specimen 10

30′ 5′

Baseline activity

$1/4^{th}$ administered activity

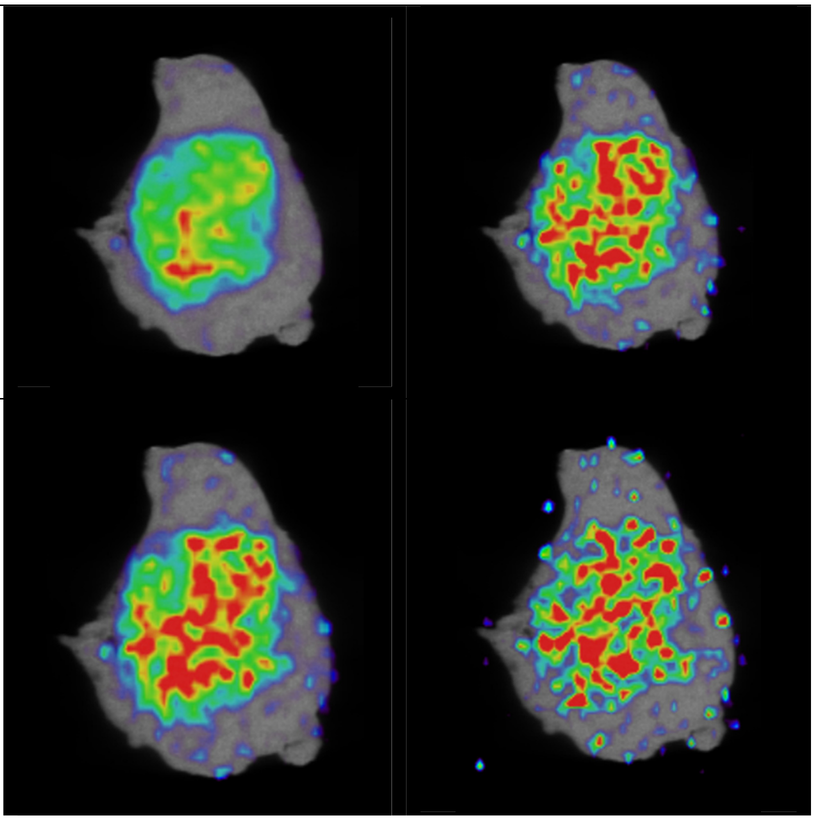

Figure S10. Original (top left) and simulated PET scans for specimen 10.

	30′	5′
Baseline activity		
1/4th administered activity		

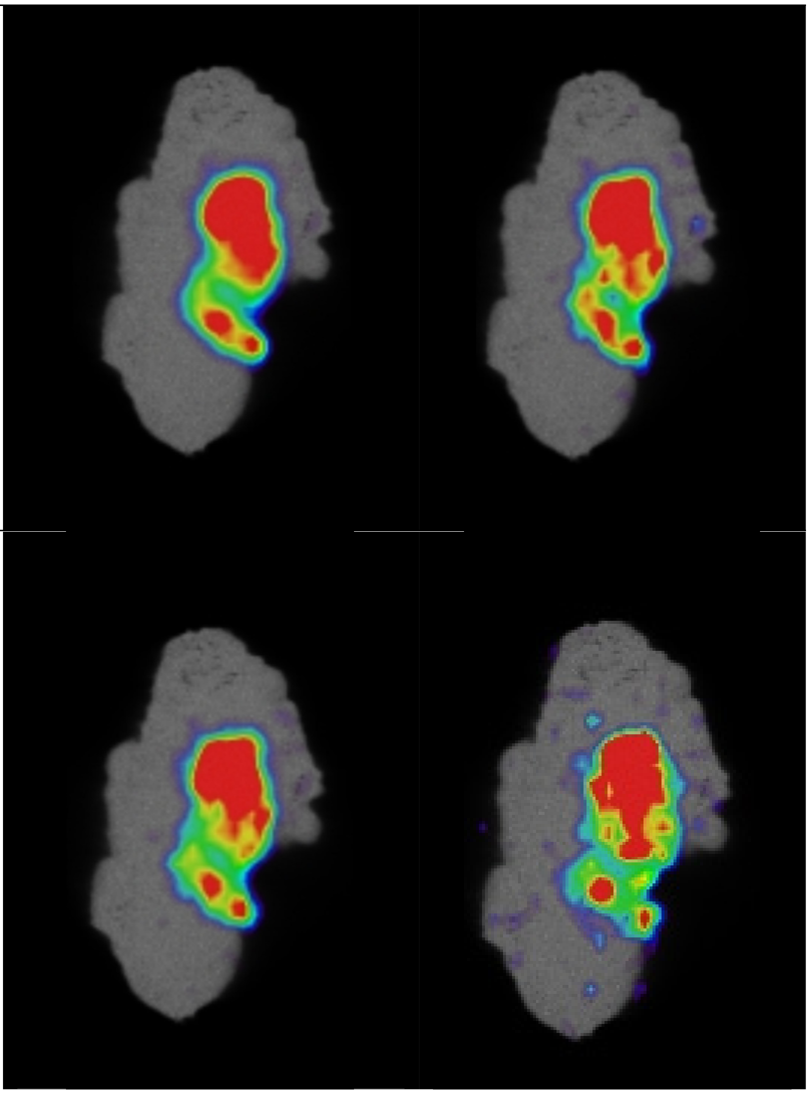

Figure S11. Original (top left) and simulated PET scans for specimen 11.
